# Two-Staged Sequential Management of Post-LASIK Ectasia: Under-Flap Corneal Cross-Linking for Stabilization Followed by Flap Surface Topography-Guided PRK for Visual Optimization

**DOI:** 10.3390/biomedicines13051258

**Published:** 2025-05-21

**Authors:** Avi Wallerstein, Brandon Bellware, Mark Cohen, Pierre Demers, Mathieu Gauvin

**Affiliations:** 1 Department of Ophthalmology and Visual Sciences, McGill University, Montreal, QC H3A 0G4, Canada; mathieu.gauvin@mcgill.ca; 2 LASIK MD, Montreal, QC H3B 4W8, Canada; bbellware@lasikmd.com (B.B.); mcohen@lasikmd.com (M.C.); pdemers@lasikmd.com (P.D.); 3 Department of Ophthalmology, Université de Sherbrooke, Sherbrooke, QC J1N 3C6, Canada; 4 Department of Electrical Engineering, École de Technologie Supérieure, Montreal, QC H3C 1K3, Canada

**Keywords:** LASIK, Coneal Ectasia, under-flap CXL, topography-guided, PRK

## Abstract

**Background/Objectives**: To evaluate the efficacy, accuracy, safety, and long-term stability of topography-guided photorefractive keratectomy (TGPRK) in eyes where post-LASIK (PLE) ectasia progression was stabilized with under-flap corneal crosslinking (ufCXL). **Methods**: This retrospective interventional case series included six eyes from five patients with PLE after microkeratome LASIK. All eyes underwent ufCXL to halt ectatic progression. A shallow TGPRK enhancement was performed on the LASIK flap surface after corneal and refractive stability was confirmed (18 months median) post ufCXL Outcome measures included uncorrected and corrected distance visual acuity (UDVA, CDVA), spherical equivalent (SEQ), refractive astigmatism, keratometry, and corneal irregularity indices over a mean follow-up of 47 months. **Results**: ufCXL stabilized ectatic progression but left residual refractive errors, limiting UDVA. TGPRK performed subsequently significantly improved UDVA, from 0.38 to 0.10 LogMAR (*p* = 0.017), and increased the LASIK efficacy index from 0.46 to 0.83 (*p* = 0.0087). Refractive astigmatism was reduced in all eyes achieving a SEQ within ±1.00 D of the target. Long-term stability was maintained, with no ectasia progression, no change in SEQ, no change in corneal irregularity indices, and no increase in maximal keratometry. **Conclusions**: TGPRK performed in ufCXL stabilized corneas can safely correct residual refractive errors, resulting in significant and sustained improvements in both refractive and visual outcomes in PLE.

## 1. Introduction

Post-laser-assisted keratomileusis (LASIK) ectasia (PLE) is a rare adverse outcome following LASIK. Characteristic signs of this complication are decreased corneal biomechanical stability, stromal thinning, keratometric inferior steepening, progressive corneal irregularity and, ultimately, irregular corneal astigmatism [1,2,3,4]. This leads to deterioration in uncorrected distance visual acuity (UDVA), corrected distance visual acuity (CDVA), and quality of vision (QoV), and can be accompanied by increased light sensitivity, glare, halos, and rarely monocular diplopia [5]. Collagen cross-linking (CXL) has shown efficacy in halting the progression of PLE, although residual refractive errors may remain [6,7,8,9,10]. The traditional epithelium-off procedure, however, is not free from complications, such as persistent epithelial defects, infection, corneal scarring and recurrent erosion, as well as an extended recovery period with pain and discomfort [11]. Epithelium-on techniques minimize recovery time and complications but have not shown the same efficacy [12,13].

A less invasive technique, termed under-flap corneal crosslinking (ufCXL) was introduced to treat early PLE before a significant change in corneal irregularity or loss of CDVA occurs. This method involves re-lifting the flap, soaking the stromal bed with riboflavin, repositioning the flap, and applying ultraviolet (UV) light. CXL therefore occurs under the flap, but not in the flap itself [14]. In a 36-month study, ufCXL was demonstrated to be safe to halt the progression of PLE with statistical maintenance of visual function, refractive astigmatism, and keratometry [15]. ufCXL is performed without combined laser ablation, and as such, visual acuity and QoV are typically only stabilized but not improved [15]. On rare occasions, ufCXL, like regular CXL, can also induce corneal changes, such as flattening, which may impact refractive and visual outcomes [16,17,18].

In post-ufCXL patients with reduced UDVA, we hypothesize that a topography-guided photorefractive keratectomy (TGPRK) enhancement can be safely performed on the LASIK flap surface once topography and refraction show stability, with central corneal thickness back to baseline levels, several months after ufCXL. The concept of performing laser ablation along with CXL in ectatic corneas is not new and is reported to be safe, although somewhat controversial [19,20]. For example, PLE and keratoconus (KC) patients undergoing same-day combined TGPRK with CXL have both demonstrated comparable stability to conventional CXL [19,20]. The literature suggests that once a flap is made, it no longer contributes to the biomechanical stability of the cornea [21]. We therefore hypothesized that the flap stroma, which did not receive crosslinking, could be safely ablated without biomechanical impact. A shallow, TGPRK ablation on the flap surface was performed to improve refraction and vision post-ufCXL. This small case series reports the visual and refractive outcomes and long-term stability of six PLE eyes that underwent TGPRK enhancement on the flap surface at least 10 months after ufCXL with stable refraction. We investigate the safety and effectiveness of this technique for improving UDVA with the aim of reducing spectacle dependence without compromising corneal stability.

## 2. Patients and Methods

This retrospective interventional case series included six eyes from five patients diagnosed with PLE after microkeratome LASIK. Staged TG PRK was performed after ectatic stabilization with ufCXL. This study was approved by the Ethics Review Board of the Canadian Ophthalmic Research Review and Ethics Compliance Team (CORRECT). All patients provided written consent for the use of anonymized data for research. The study was conducted in accordance with the tenets of the Declaration of Helsinki.

### 2.1. Standardized Identification of Post-Lasik Ectasia

In contrast to previous CXL studies where corneal ectasia is commonly treated at an advanced stage with CDVA significantly reduced [6], the current ufCXL technique aims to treat milder ectasia before a significant reduction in CDVA. Inclusion criteria were eyes that had myopic LASIK with early to moderate post-LASIK ectasia, defined as presenting with the new onset of progressive manifest refraction cylinder up to 2.50 diopters (D), a new decrease in uncorrected distance visual acuity (UDVA) not greater than 20/100, corrected distance visual acuity (CDVA) of 20/25 or better, and new topographic irregular astigmatism and/or inferior steepening consistent with post-LASIK ectasia, as previously reported [14]. All cases were sent to a consultant group of five highly experienced surgeons who confirmed the diagnosis of PLE using previously published standardized criteria for requiring ufCXL intervention. These included topographical changes consistent with PLE with a decrease in UDVA and/or CDVA, and/or a decrease in quality of vision symptoms as subjectively described by the patient as causing new or worsening halos, glare, ghosting, or shadowing [14].

### 2.2. ufCXL Technique

As previously described [15], the flap edge was identified at the slit lamp and a small 1- to 2-clock hour opening was created. Under the excimer laser microscope (EX500, Alcon Laboratories, Fort Worth, TX, USA), the entire flap was lifted. Under-flap stromal bed thickness needed to be 300 µm or greater prior to riboflavin administration. A circular sponge soaked in 0.25% isotonic riboflavin was placed on the stromal bed and rewetted at 60 s intervals for 5 min. Care was taken not to soak the flap with riboflavin. After 5 min, the bed was dried and excess riboflavin was removed with a surgical spear. The flap was replaced with minimal irrigation. Ultraviolet light (CCL-VARIO Cross-linking Radiation system; Peschke Trade GmbH, Hünenberg, Switzerland) was applied for 5 min at 18 mW/cm^2^, resulting in a total irradiation dose of 5.4 J/cm^2^ at the corneal surface. Lubricating or topical anesthetic drops were applied every 30 s during light exposure. The flap was assessed at the slit lamp prior to discharge. No bandage contact lens was applied.

### 2.3. Post-ufCXL Regimen

Gatifloxacin 0.3% (Zymar; Allergan, Inc., Irvine, CA, USA) four times a day for 5 days and prednisolone acetate 1% (Pred Forte; Allergan, Inc.) every hour for day 1, every 2 h for day 2, and four times a day for days 3, 4, and 5 were given postoperatively. Refresh tear drops were used every half hour for day 1, hourly for day 2, and then four times a day for 5 days. Strict instructions to avoid eye rubbing and squeezing of the eyelids were given to all patients. Patients wore protective sunglasses for 48 h postoperatively.

### 2.4. Topography-Guided PRK

Once a minimum stabilization period of at least 10 months post-ufCXL was confirmed, based on stable topography and refraction, patients with residual refractive errors were considered for TGPRK enhancement. Preoperatively, a minimum of 4 high-quality reproducible scans were obtained using Allegro Topolyzer Vario (Alcon Laboratories, Inc., Fort Worth, TX, USA) along with an additional 2 scans using Orbscan-IIz (Bausch & Lomb, Inc., Vaughan, ON, Canada). The corneal astigmatism magnitude and axis derived from these scans were averaged according to a previously published protocol [22]. The calculated astigmatism magnitude and axis were then inputted into the Contoura surgical planning software v.1.76r76 (Alcon Laboratories, Inc., Fort Worth, TX, USA). Before proceeding with excimer ablation, the Orbscan elevation map was compared with the HOA ablation depth planning software map to ensure concordance, v.1.76r76. Excimer ablation of the anterior corneal HOAs and calculated refraction was undertaken. An optical zone size of 5.0 mm or 5.5 mm was selected depending on the topographic cone peak location within or outside of the central 3 mm ring, respectively.

Following the application of topical proparacaine hydrochloride 0.5% anesthetic drops (Alcaine, Alcon Laboratories, Inc.), a 6.5 mm zone of the epithelium was partially removed using a 40 μm phototherapeutic keratectomy (PTK). A small myopic ablation (approximately −0.75 D) was performed to offset any hyperopic treatment effect from PTK. TGPRK was then executed with the WaveLight EX500 excimer laser, ablating flap stroma (which had not been crosslinked during ufCXL). Mitomycin-C (0.02%) was applied to the stromal bed for 2 min to mitigate postoperative haze formation. The postoperative regimen included the administration of Zymar 4 times a day for 7 days, Acuvail once per day for 2 days, and Maxidex 4 times a day for 8 weeks. The bandage contact lens was removed on the fifth day.

### 2.5. Data and Statistical Analysis

Data from ophthalmic examinations pre-LASIK, pre-ufCXL, post-ufCXL, pre-TGPRK and at 1, 3, 6, 12, 24, and 36 months and later time points post-TGPRK were collected for analysis, including slit-lamp examinations, manifest refraction sphere, cylinder, and spherical equivalent (SEQ), uncorrected and corrected distance visual acuity (UDVA and CDVA, respectively), maximum keratometry (Kmax), central pachymetry, corneal irregularity indices, and presence of haze and/or other complications at the slit lamp. Keratometry and corneal irregularity indices were obtained with the Orbscan IIz system (Bausch & Lomb) on each eye and time point. Failure of ufCXL or PLE progression post-TGPRK was defined as an increase in Kmax of 1.00 D or greater over the pre-ufCXL value, similar to previous CXL studies [15,22]. Data were analyzed using MATLAB R2024B (MathWorks, Inc., Natick, MA, USA). For normally distributed variables, paired *t*-tests were used to compare pre- and post-TGPRK outcomes, while the two-sample Wilcoxon signed-rank test was applied for non-normally distributed data. The Kolmogorov–Smirnov test was employed to assess the normality of data distributions. Statistical significance was defined as a *p*-value less than 0.05.

## 3. Results

Data were available for 6 eyes of 5 patients who underwent previous microkeratome LASIK, presented with early PLE, underwent ufCXL, followed by a TGPRK enhancement once the topography and refractive measurements had shown stability. The average age at the last follow-up was 47 ± 11 years. The average time from LASIK to ufCXL was 115 ± 62.5 months, with an average time from ufCXL to TGPRK enhancement of 23 ± 17 months. The average last follow-up time post-TGPRK was 47 ± 27 months.

### 3.1. Visual Outcomes

**Figure 1:**
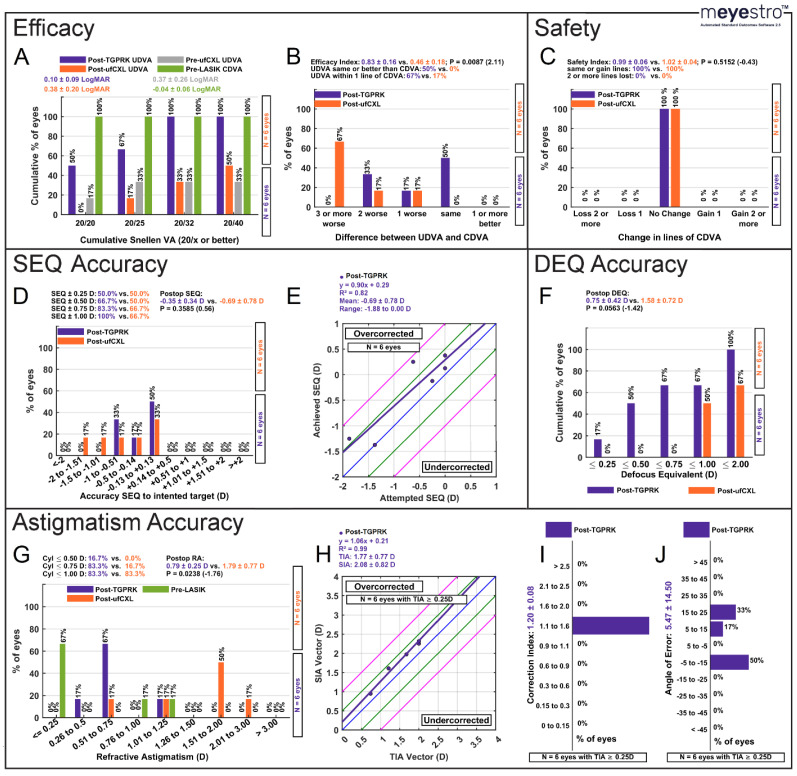
(**A**) Cumulative postoperative Snellen UDVA compared with preoperative (**B**) Difference in Snellen lines between postoperative UDVA and preoperative CDVA. (**C**) Change in postoperative Snellen lines of CDVA compared with preoperative CDVA. (**D**) Postoperative spherical equivalent (SEQ) histogram. (**E**) The blue line indicates attempted = achieved, green lines indicate ±0.50 D, and pink lines indicate ±1.00 D. The purple line is the linear fitting between the attempted and achieved SEQ. (**F**) Cumulative postoperative defocus equivalent (DEQ) histogram. DEQ is defined as the summation of the absolute value of the SEQ and half the absolute value of the astigmatism. (**G**) Postoperative refractive astigmatism compared to preoperative refractive astigmatism. (**H**) The blue line indicates TIA = SIA, green lines indicate ±0.50 D, and pink lines indicate ±1.00 D. The purple line is the linear fitting between the TIA and SIA vectors. (**I**) Postoperative Correction Index histogram. (**J**) Postoperative Angle of Error histogram. **Abbreviations**: CDVA = corrected distance visual acuity, D = diopters, cyl = cylinder, DEQ = defocus equivalent, SEQ = spherical equivalent, SIA = surgically induced astigmatism; TIA = target-induced astigmatism, UDVA = uncorrected distance visual acuity.

Monocular UDVA was 0.38 ± 0.20 LogMAR post-ufCXL (last post-ufCXL postop immediately before TGPRK enhancement) and statistically improved to 0.10 ± 0.09 LogMAR (approximately 3 lines improvement) post-TGPRK enhancement (*p* = 0.0170; Figure 1A). There was a non-statistically significant increase in the percentage of eyes achieving a cumulative monocular Snellen UDVA of 20/25 post-TGPRK versus post-ufCXL alone (67% vs. 17%, *p* = 0.0930; Figure 1A). 100% of eyes achieved a cumulative monocular Snellen UDVA of 20/40 post-TGPRK versus only 50% pre-TGPRK (*p* = 0.0555; Figure 1A). Prior to TGPRK, only 20% of patients achieved a binocular UDVA of 20/20, while the remaining 80% achieved 20/40 (average: 0.24 ± 0.13 LogMAR). In contrast, at 47 months post-TGPRK, 60% of patients achieved 20/20, with the remaining patients achieving binocular UDVA of 20/25 and 20/30, respectively (average: 0.06 ± 0.09 LogMAR). No patient had a UDVA of 20/40 at 47 months post-TGPRK compared to 80% pre-TGPRK. This demonstrates greatly improved and sustained functional uncorrected vision even four years after TGPRK intervention.

Prior to TGPRK intervention, PLE resulted in a decreased LASIK efficacy index of 0.46 ± 0.18, which was substantially improved to 0.83 ± 0.16 post-TGPRK (*p* = 0.0087; *ES*: 2.11; Figure 1B). Improvements were also apparent when comparing the difference in Snellen lines of post-ufCXL UDVA to pre-LASIK CDVA (67% with 3 or more lines worse) and post-TGRPK UDVA to pre-LASIK UDVA (0% with 3 or more lines worse, *p* = 0.0009; Figure 1B). Post-TGPRK, 67% of eyes had UDVA within one line of pre-LASIK CDVA, compared to 17% of eyes post-ufCXL alone (*p* = 0.0930; Figure 1B).

### 3.2. Safety

The safety index was unchanged post-ufCXL to post-TGPRK (1.02 ± 0.04 vs. 0.99 ± 0.06, *p* = 0.5152; *ES*: −0.43; Figure 1C), with all eyes having the same Snellen CDVA of 20/20 or better 23 months post-ufCXL and 47 months post-TGPRK (Figure 1C).

### 3.3. SEQ and Defocus Equivalent Accuracy

**Figure 2:**
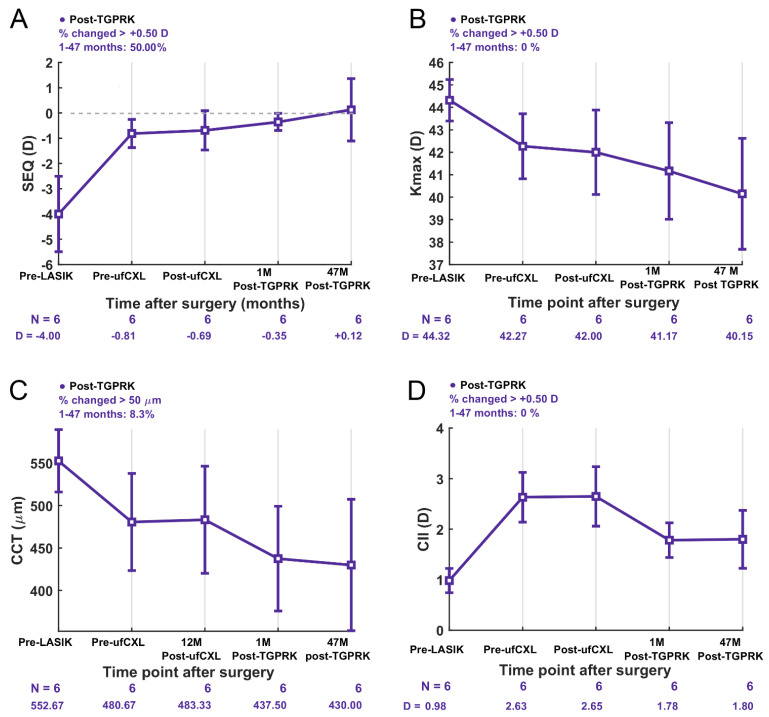
Average (**A**) SEQ, (**B**) maximum keratometry, (**C**) central corneal thickness, and (**D**) corneal irregularity index at 3 mm pre-LASIK, pre-ufCXL, post-ufCXL, as well as 1 and 47 months post-TGPRK. Error bars show ±1 standard error. CCT = central corneal thickness; CII = corneal irregularity index; D = diopters; Kmax = maximum keratometry; LASIK = laser in situ keratomileusis; SEQ = spherical equivalent; TGPRK = topography-guided photorefractive keratectomy; ufCXL = under-flap stromal bed corneal cross-linking.

SEQ was −0.69 ± 0.78 D post-ufCXL and clinically significantly improved to −0.35 ± 0.34 D 1 month post-TGPRK enhancement (*p* = 0.3585; *ES*: 0.56; Figure 1D). A total of only 50%, 66.7%, and 66.7% of eyes were within ±0.50, ±0.75 and ±1.00 D of intended correction post-ufCXL compared to 66.7%, 83.3% and 100% post-TGPRK (Figure 1D). The 1-month predictability of TGPRK enhancements was therefore satisfactory with a R^2^ value of 0.82 (Figure 1E). Between 1 month and 47 months post-TGPRK the SEQ remained stable, with a final value of +0.12 ± 1.23 D (*p* = 0.3434; *ES*: 0.43; Figure 2A).

There was a non-significant but clinically meaningful trend (effect size = −1.42) of improved average defocus equivalent post-TGPRK compared to post-ufCXL (0.75 ± 0.42 vs. 1.58 ± 0.72; *p* = 0.0563, Figure 1F), with significantly more eyes with a DEQ of 0.75 D or less post-TGPRK compared to pre-TGPRK (67% vs. 0%, *p* = 0.0186, Figure 1F).

### 3.4. Refractive Astigmatism Accuracy

TGPRK enhancements resulted in a statistically significant reduction in the average refractive astigmatism post-TGPRK compared to post-ufCXL alone (0.79 ± 0.25 D vs. 1.79 ± 0.77 D; *p* = 0.0238; *ES*: −1.76; Figure 1G). There were significantly more eyes with refractive cylinder ≤ 0.75 D post-TGPRK compared to post-ufCXL (83.3% vs. 16.7%, *p* = 0.0272; Figure 1G). The target-induced astigmatism versus surgically induced astigmatism predictability of the TGPRK intervention was excellent, with R^2^ values of 0.99, respectively (Figure 1H). The correction index was 1.20 ± 0.08 (Figure 1I), and the angle of error was 5.57 ± 14.50 (Figure 1J). Between 1 month and 47 months post-TGPRK the refractive cylinder remained stable, with a final value of −0.83 ± 0.70 D (*p* = 0.8945; *ES*: 0.06).

### 3.5. Stability of SEQ, Keratometry, Corneal Thickness, and Corneal Irregularity

Long-term SEQ stability analysis showed a slight hyperopic shift averaging +0.47 D from 1 month to 47 months post-TGPRK, with 50% of eyes a change greater than 0.50 D (Figure 2A), a direct result of the corresponding keratometric flattening (Figure 2B). This hyperopic shift contributed to a non-statistically significant change from −0.35 ± 0.34 D 1 month post-TGPRK to +0.12 ± 1.23 D 47 months post-TGPRK (*p* = 0.3434; Figure 2A).

Long-term keratometry analysis demonstrated a consistent decrease in Kmax (flattening) across all eyes post-ufCXL that continued post-TGPRK. Specifically, Kmax decreased slightly from 42.00 ± 1.88 D post-ufCXL to 41.17 ± 2.15 D 1 month post-TGPRK due to the excimer laser ablation, and continued to non-statistically decrease to 40.15 ± 2.47 D at 47 months post-TGPRK, likely due to the ongoing post-ufCXL flattening process (*p* = 0.1005; Figure 2B). No eyes experienced a Kmax increase greater than +0.50 D from 1 month to 47 months post-TGPRK, providing preliminary evidence that PLE did not progress despite the TGPRK intervention (Figure 2B).

Long-term pachymetry analysis demonstrated statistically stable central corneal thickness, from 437.5 ± 61.7 µm at 1 month post-TGPRK to 430.00 ± 77.4 µm at 47 months post-TGPRK (*p* = 0.7158; Figure 2C). One eye (8.3%) showed a change greater than 50 µm, attributed to CXL-induced thinning, a known potential effect of CXL (Figure 2C).

Corneal irregularity analysis demonstrated an impressive reduction in the corneal irregularity index (CII) in the central 3 mm zone from 2.65 ± 0.58 D post-ufCXL to 1.78 ± 0.34 at 1 month post-TGPRK (*p* = 0.0057, Figure 2D). This reduction was attributed to the topography-guided excimer laser ablation of PLE-induced higher-order aberrations. The improvement remained statistically stable, with a CII of 1.80 ± 0.57 D at 47 months post-TGPRK (*p* = 0.9324, Figure 2D). CII at 5 mm was also maintained 47 months post-TGPRK (3.02 ± 0.47 vs. 2.92 ± 0.59; *p* = 0.6142).

Topographical findings are illustrated with the keratometric maps, shown at various time points (before ufCXL, after ufCXL, and at the latest available post-TGPRK follow-up) for the entire cohort in Supplementary Figure S1 (available in the online version of this article).

### 3.6. Complications

Early after TGPRK 1 eye had significant edema and corneal haze that had resolved shortly after, and 1 eye had irregular central epithelium. These complications remained non-visually significant after resolution by the last post-TGPRK visit. From ufCXL to the last post-TGPRK visit two patients had progressive corneal Kmax flattening greater than 1.00 D, due to the initial ufCXL intervention, that eventually stabilized. These were non-visually significant with both eyes still having 20/20 or better monocular CDVA and 20/20 bilateral UDVA. No epithelial ingrowth, haze, or other complications were noted.

## 4. Discussion

While PLE is rare, with incidence ranging from 0.04 to 0.6%, its progression can result in biomechanical corneal decompensation, stromal thinning, keratometric inferior steepening, progressive corneal irregularity with irregular corneal astigmatism, and ultimately, significant quality of vision and quality of life deterioration [1,2,3,4]. CXL has shown excellent efficacy and safety in halting the progression of PLE as well as keratoconus [6,7,8,9,10]. However, the traditional epithelium-off procedure is not free from complications, such as persistent epithelial defects, infection, corneal scarring and recurrent erosion, as well as an extended recovery with pain and discomfort [11].

Under-flap CXL has been recently introduced as a less invasive technique to halt and stabilize early PLE. It is performed in early cases of PLE, when cornea biomechanics and vision are only minimally affected, with a small amount of irregular astigmatism and before any significant CDVA loss occurs [14]. More advanced cases of ectasia, in the author’s practice, are treated with same-day sequential TGPRK with CXL [22]. In ufCXL, the LASIK flap is lifted, riboflavin is applied to the stromal bed, the flap is repositioned, and UV light is administered to the corneal surface, thereby achieving crosslinking exclusively within the stromal bed beneath the flap [14]. The epithelium remains intact and the CXL effect is achieved deeper in the corneal stroma below the flap since the epithelium stays on. ufCXL has fewer complications than standard epithelium-off CXL, minimizing dry eye, pain, and haze, with minimal quicker recovery [14].

In a published series of 20 eyes treated with ufCXL, a 36-month follow-up demonstrated stable safety, visual outcomes, refractive astigmatism and maximum keratometry, with preservation of normal endothelial cell density, and a consistently deep stromal demarcation line [15]. No eye showed progression of more than 1.00 diopter in maximum keratometry 3 years after treatment, highlighting ufCXL potential to halt early PLE progression [15].

Although ufCXL effectively stabilizes early PLE, it does not correct the residual refractive errors already induced by the ectasia [15]. Furthermore, the unpredictable corneal flattening associated with CXL, and the variable epi remodeling coupled with regression over time, can worsen these residual errors after ufCXL [23,24]. Further interventions to improve UDVA and overall quality of vision once ufCXL has biomechanically strengthened the corneal stroma below the flap and stabilized PLE remain warranted for certain patients. The current preliminary case series explored the efficacy, safety, accuracy, and stability of performing topography-guided PRK once PLE stabilization was confirmed 18 months (median) after ufCXL. The aim was to reduce refractive error and to regularize the corneal surface to improve UDVA and QoV without compromising the long-term stability achieved by ufCXL.

The concept of same-day sequential CXL and TGPRK for PLE and keratoconus is well-established, with numerous short- and long-term studies demonstrating its safety and efficacy [19,20,22,24]. Protocols have shown improvements in UDVA and QoV, and studies on ectatic eyes treated with simultaneous CXL and TGPRK report stability comparable to conventional CXL alone [19,20,22]. The current approach differs in several ways. TGPRK is deferred until ufCXL corneal stabilization is confirmed. The ufCXL technique also allows the flap to remain untouched by cross-linking and preserved for future ablation. This contrasts with traditional staged CXL and TGPRK in separate sessions where cross-linked tissue gets ablated. Simultaneous same-day and traditional separate session ablation protocols also do not aim for full visual rehabilitation but rather regularize the cornea and minimize tissue removal. Because with ufCXL, PLE is detected early, prior to significant corneal distortion or induced astigmatism, the required correction is minimal, resulting in less tissue ablation.

The current study’s post-ufCXL TGPRK takes advantage of the LASIK flap stroma for excimer ablation. Because ufCXL does not cross-link the flap, this stromal flap tissue remains available for refractive correction and corneal regularization. Ablating non-crosslinked tissue reduces variability in laser ablation compared to ablating crosslinked tissue [25]. Furthermore, once a LASIK flap is created, it contributes minimally to corneal biomechanics [21]. Performing a shallow surface ablative procedure of the non-crosslinked flap tissue in these stabilized ectatic eyes is therefore inherently safer. By confining the ablation to the flap stroma, biomechanical risk is minimized. Moreover, performing the TGPRK several months after ufCXL allows clinicians to confirm corneal stability before correcting PLE-induced and CXL-induced changes, with the intent of improving UDVA and overall visual quality. 

We report long-term, 4-year stability and no progression of ectasia following TGPRK treatment in six post-ufCXL eyes with mild to moderate PLE. At 1 month post-TGPRK, there was already a statistically significant increase in the percentage of eyes achieving monocular cumulative Snellen UDVA of 20/20 and 20/25 or better compared to pre-TGPRK with patients gaining an average 0.28 LogMAR of UDVA. Still four years after TGPRK, patients gained an average of 2 lines of functional UDVA compared to pre-TGPRK, with no patients losing lines of UDVA nor CDVA. This demonstrates improved and sustained functional uncorrected vision maintained at an average of 4 years after the second intervention. Although visual improvements of an average gain of 2 lines in UDVA were seen long-term, not all patients reached 20/20 UDVA. Refractively, there was a clinically meaningful trend of reduced average defocus equivalent post-TGPRK, with significantly more eyes achieving a defocus equivalent of 0.75 D or less post-TGPRK compared to pre-TGPRK (67% vs. 0%). Overall, 4 years after TGPRK, none of the eyes showed an increase in Kmax (no steepening) with stable SEQ, cylinder, corneal thickness and corneal irregularity index. Although, introducing a TGPRK 12 months or more after ufCXL might introduce variability due to prior or still ongoing long-term ufCXL effects, as well as the difference in biomechanics in PLE corneas [16,25,26], the attempt to achieved refractive astigmatism predictability of TGPRK enhancement post-ufCXL was reliable with R^2^ or 0.99. The TIA to SIA graphs, however, showed a slight overcorrection of astigmatism (CI = 1.20) suggesting the astigmatism should be slightly undercorrected by 20% upon treating those eyes with TGPRK post-ufCXL.

With routine, annual post-LASIK follow-up, PLE can be identified before significant irreversible changes occur and ectatic progression is halted with ufCXL. Once corneal stability is confirmed, a surface TGPRK is performed to correct residual refractive error and regularize the cornea. This novel technique introduces a paradigm shift in the traditional view of PLE from a debilitating complication to a manageable treatable condition. In select cases, visual outcomes may reach a level where corrective eyewear is no longer required.

### Limitations

The small sample size reflects the low incidence of PLE and may limit the statistical power of this study. This preliminary study should therefore be interpreted as proof-of-concept rather than definitive evidence of safety. Statistically significant improvements were found from pre- to post-TGPRK. Since only eyes with relatively mild to moderate PLE were included, the effectiveness of long-term sequential ufCXL and TGPRK in more advanced PLE cases remains unknown. Larger prospective studies are needed to confirm these findings and report more variables such as higher order aberration, QoV, corneal epithelium mapping looking at epithelial irregularity, and contrast sensitivity.

## 5. Conclusions

The current findings suggest that TGPRK performed in ufCXL stabilized PLE corneas, can safely correct residual refractive errors, resulting in significant and sustained improvements in both refractive and visual outcomes. No ectatic progression was observed during long-term follow-up. While larger randomized studies are needed to validate these results and define optimal treatment parameters, this study provides the first evidence that TGPRK enhancement, performed at least one year after post-ufCXL stability, can be an option for improving visual function in eyes with mild to moderate PLE eyes.

## Data Availability

The original contributions presented in the study are included in the article or Supplementary Materials (Supplementary Figure S1), further inquiries can be directed to the corresponding author.

## Supplementary Materials

The following supporting information can be downloaded at https://www.mdpi.com/article/10.3390/biomedicines13051258/s1, Figure S1: Keratometric maps before ufCXL, after ufCXL (immediately before TGPRK) and after TGPRK. Kmax = maximum keratometry; TGPRK = Topography-guided photorefractive keratectomy; ufCXL = under-flap stromal bed corneal cross-linking.
