# Supplementary material for: Two-Staged Sequential Management of Post-LASIK Ectasia: Under-Flap Corneal Cross-Linking for Stabilization Followed by Flap Surface Topography-Guided PRK for Visual Optimization
Source: Biomedicines. 2025 May 21;13(5):1258. doi: 10.3390/biomedicines13051258 (PMC12108988; doi:10.3390/biomedicines13051258)
Supplement: Supplementary file 1 [file biomedicines-13-01258-s001.zip › biomedicines-3622751-supplementary.pdf]

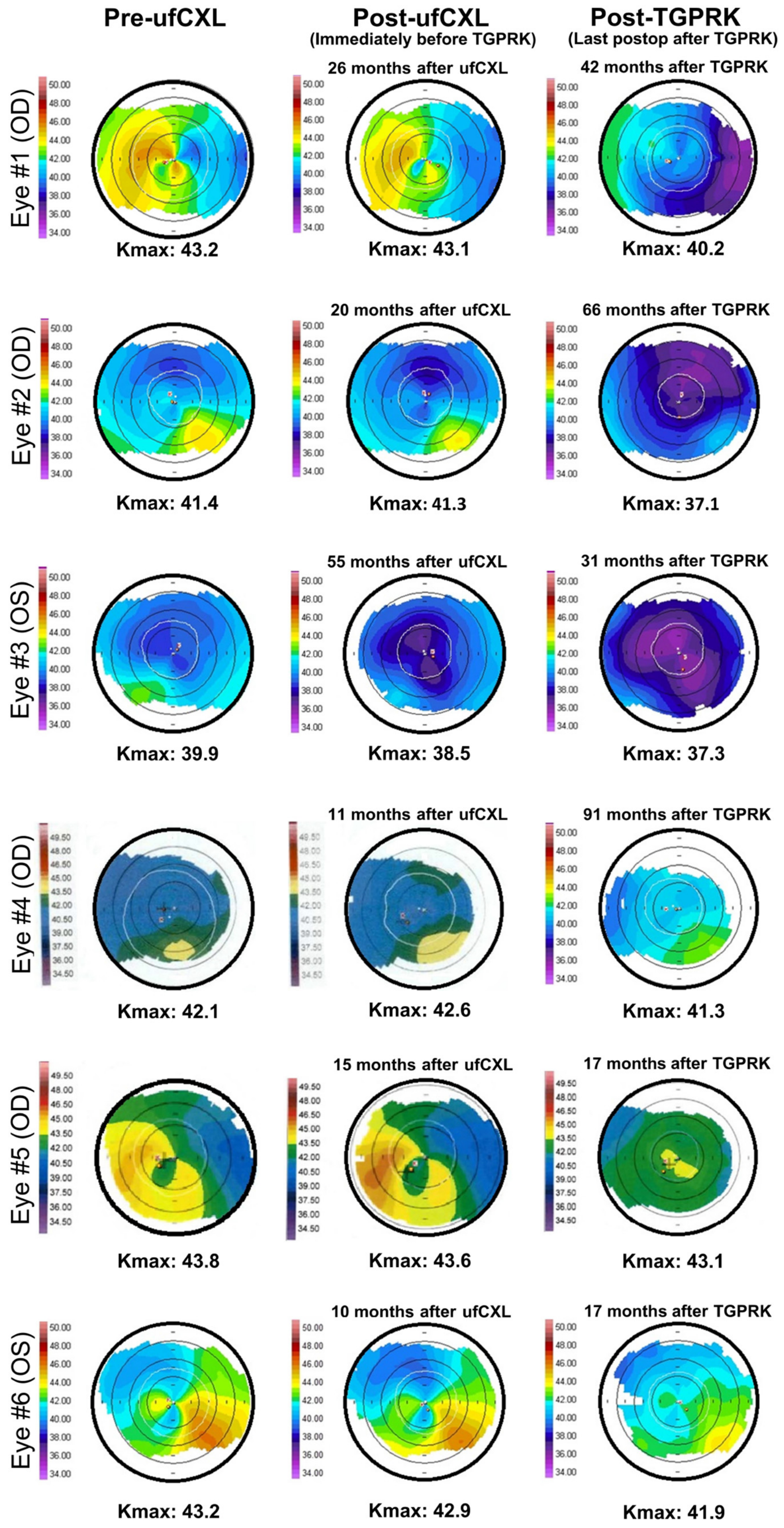

Figure S1. Keratometric maps before ufCXL, after ufCXL (immediately before TGPRK) and after TGPRK. Kmax = maximum keratometry; TGPRK = Topography-guided photorefractive keratectomy; ufCXL = under-flap stromal bed corneal cross-linking.
